# Characterization and phylogenetic analysis of the chloroplast genome of *Vicia kulingana* (Fabaceae)

**DOI:** 10.1080/23802359.2025.2485168

**Published:** 2025-03-31

**Authors:** Xingxing Chen, Zhuo Cheng, Zhongxin Duan, Dan Xi, Binsheng Luo

**Affiliations:** a Lushan Botanical Garden, Jiangxi Province and Chinese Academy of Sciences, Lushan, China; b College of Life and Environmental Sciences, Minzu University of China, Beijing, China

**Keywords:** Edible and medicinal plant, genetic diversity, monophyletic group, *Lens*

## Abstract

*Vicia kulingana* L. H. Bailey 1920, a famous edible and medicinal plant, had its chloroplast genome sequenced in this study to determine its phylogenetic relationship with other related species. The research findings are as follows: 1. The chloroplast genome of *V. kulingana* was 125, 696 bp long and lacked an inverted repeat unit. 2. The genome contains a total of 102 genes, including 69 protein-coding genes, 28 tRNAs, and 5 ribosomal RNA. 3. Phylogenetic analysis revealed that *V. kulingana* and other three *Vicia* species are genetically closely related and are located on the same branch of the phylogenetic tree, indicating a sister group relationship among them. Our results support that *Lens* was treated as a synonym of *Vicia*. This study provides a foundation for the identification, classification, and exploration of genetic diversity within the *Vicia* genus.

## Introduction

*Vicia* L., a genus belonging to the Fabaceae family, includes approximately 180 to 210 species. These plants are predominantly found in temperate regions of the Northern Hemisphere, as well as in temperate areas of South America and tropical Africa (Jo et al. 2022). *Vicia* species are of considerable economic importance, with various uses, including green manure, cover crops, forage, and honey production, in addition to being an essential genetic resource (Li et al. 2020).

**Figure 1.**
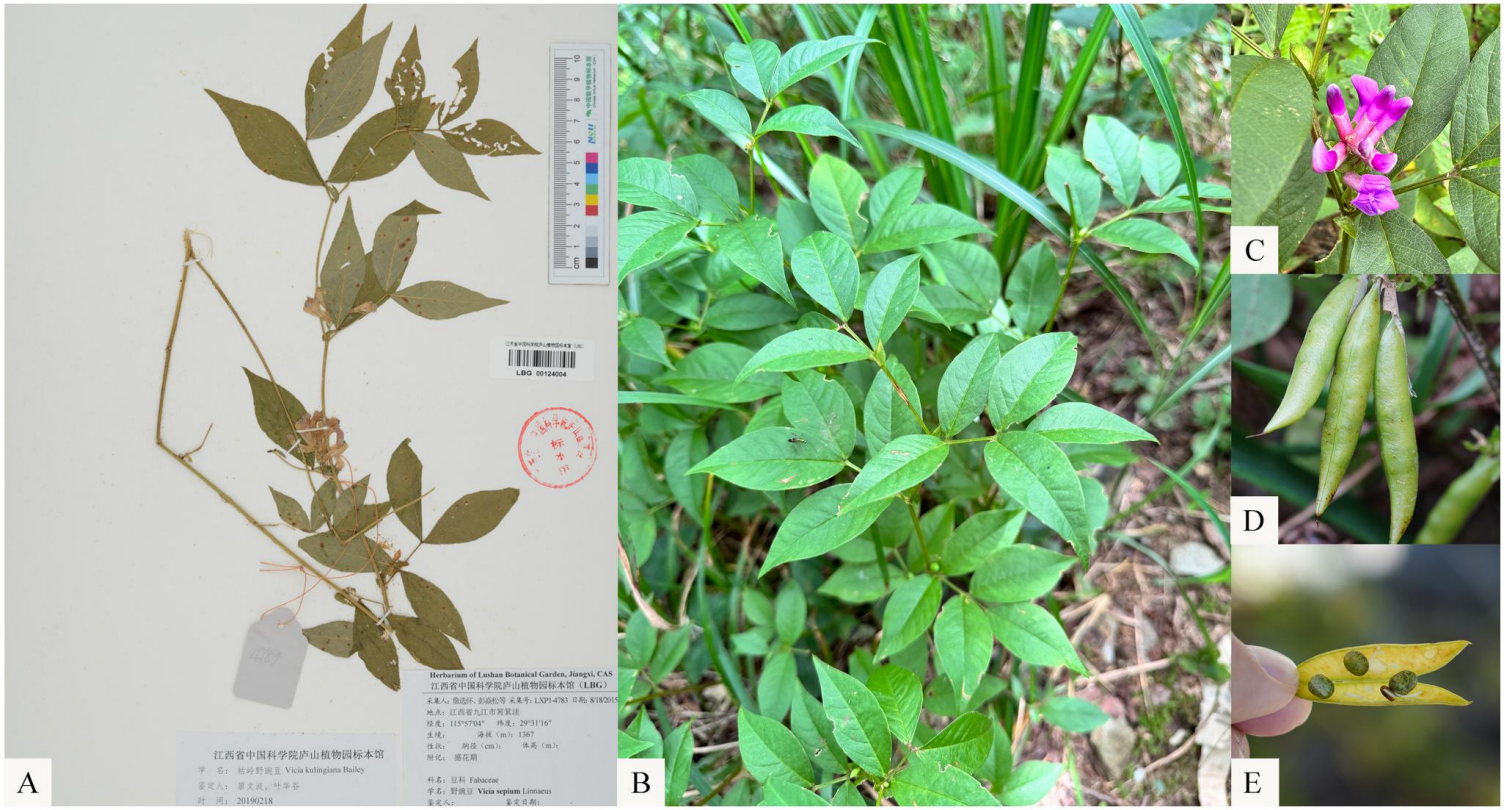
A. Voucher specimen of *V. kulingana*; B. Plant image of *V. kulingana*; C. Flowers; D. Fruits; E. Seeds. Binsheng Luo photographed these photos at Lushan Botanical Garden in Jiangxi. The distinction between this species and other *Vicia* species is that bracts are persistent at the base of pedicels.

*Vicia kulingana* L. H. Bailey, a species within the *Vicia* genus, is native to China, especially in the eastern parts of the country (Liu et al. 2015). The species is named after Guling Town (also known as Kuling), where its type specimen was first collected, located in the famous Mount Lu in Jiangxi Province (Figure 1). *V. kulingana* is adaptable to various habitats, such as valley bamboo forests, wetlands, grasslands, and sandy soils, typically flourishing at altitudes ranging from 200 m to 1200 m. The flowering season extends from April to August, while fruiting occurs between June and September (Nigel, 1993). Research conducted in the Lu Shan area has highlighted the young leaves of *V. kulingana* as a highly regarded wild vegetable in local cuisine, a practice unique to this region. This preference is linked to the plant’s distinctive aroma, exceptional nutritional content, and pleasant flavor, as recorded in traditional botanical knowledge (Soyolt et al. 1998). In addition, *V. kulingana* is recognized as a high-quality forage plant with considerable prospects for economic value (Duan et al. 2024).

Previous research supported the traditional uses of *V. kulingiana*’s and highlighted its potential as a valuable food source, encouraging further research on its food applications. However, the chloroplast genome of *V. kulingiana* has not been reported.

## Materials and methods

The total genomic DNA was extracted from dried leaves collected from Guling Township, Lushan City (Jiangxi, China, E 115°57′, N 29°31′). The plant voucher herbarium specimen (Accession number: EB20230508001; contact person: Binsheng Luo; email: luobins@lsbg.cn) was deposited at the Herbarium of Lushan Botanical Garden (http://www.lsbg.cn). The total genomic DNA was extracted from the fresh leaves using the modified CTAB method (Doyle and Doyle 1987), and libraries were prepared using the TruSeq^™^ Nano DNA Sample Prep Kit (Illumina, Nanjing, CN). Genomic paired-end sequencing was performed on the Illumina Novaseq 6000 platform, generating approximately 10 GB of data. The chloroplast genome was assembled and analyzed using the NOVOPlasty-4.3.1 program (Dierckxsens et al. 2017). Annotation was performed with CPGView (http://www.1kmpg.cn/cpgview/) to determine the initial location of the chloroplast genome and the IR region and to annotate the genes (Liu et al. 2023), with the chloroplast genome of *Vicia sepium* (NC039595) serving as a reference. The comments were manually proofread to identify any errors, and the reference used was Zhou et al. (2021). The final chloroplast genome of *V. kulingana* was deposited into NCBI GenBank under the accession number PQ576733.

Fifty-six single-copy protein-coding genes (PCGs) were extracted from 27 chloroplast sequences using the PhyloSuite_v1.2.3 software (Zhang et al. 2020; Xiang et al. 2023). They were aligned using the MAFFT algorithm (Katoh et al. 2019). All these single gene alignments were concatenated to create a document for phylogenetic analyses. The best-fit model, TVM+F + R3, was determined using the Bayesian information criterion (BIC) with the ModelFinder2 program (Kalyaanamoorthy et al. 2017). To determine its phylogenetic position, a maximum likelihood (ML) tree was constructed by IQ-TREE analysis was performed with MrBayes based on the complete chloroplast genome sequences of 8 other *Vicia* species and 11 *Vicieae* species through PhyloSuite_v1.2.3 software. Phylogenetic trees were visualized, rooted with *Caragana sinica* and *Astragalus membranaceus*, and edited using the online tool Interactive Tree of Life (https://itol.embl.de). *C. sinica* and *A. membranaceus* also belong to Fabaceae, which are suitable as out groups because they are close to *Vicia* in their phylogenetic properties.

## Results

**Figure 2.**
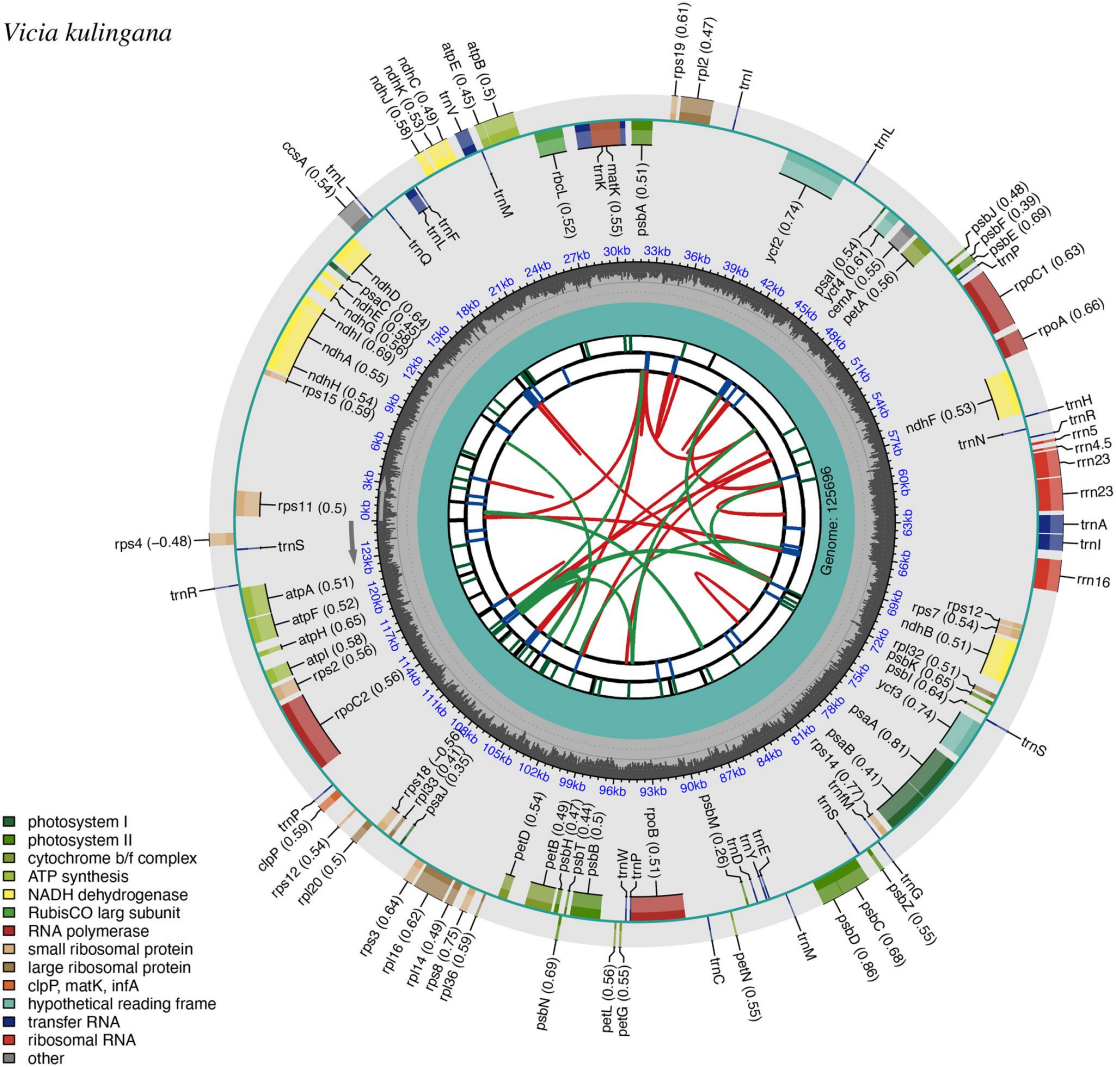
Schematic map of overall features of the *V. kulingana* chloroplast genome (genes drawn outside the outer circle are transcribed clockwise, and those inside are transcribed counter-clockwise. Genes belonging to different functional groups are color-coded. The different colored legends in the bottom left corner indicate genes with different functions. From the center outward, the first track shows the dispersed repeats. The dispersed repeats consist of direct (D) and palindromic (P) repeats, connected with red and green arcs. The optional codon usage bias is displayed in the parenthesis after the gene name.

The complete chloroplast genome of *V. kulingana* was composed of 125, 696 base pairs (bp) and lacked an inverted repeat unit, with an average depth of 2087.52 X (Figure S1). The overall GC content is 34.99%. The plastome contains a total of 102 genes, including 69 protein-coding genes (PCGs), 28 tRNAs, and 5 ribosomal RNAs (rRNAs). Furthermore, 16 genes in the chloroplast genome of *V. kulingana* contained introns. Among them, *rps12*, *ndhA*, *ndhB*, *rpl2*, *rpl16*, *petD*, *petB*, *atpF*, *rpoC1*, *clpP*, *rtrn*K-UUU, *trn*V-UAC, *trn*A-UGC, *trn*I-GAU, and *trn*L-UAA contained a single intron, whereas *ycf3* had two introns (Figure S2). The consensus phylogenetic tree is reconstructed by maximum likelihood (ML) and Bayesian inference (BI) analysis based on 56 protein-coding sequences (CDS) of 27 species, with *C. sinica* and *A. membranaceus* as outgroups (Figure 2).

**Figure 3.**
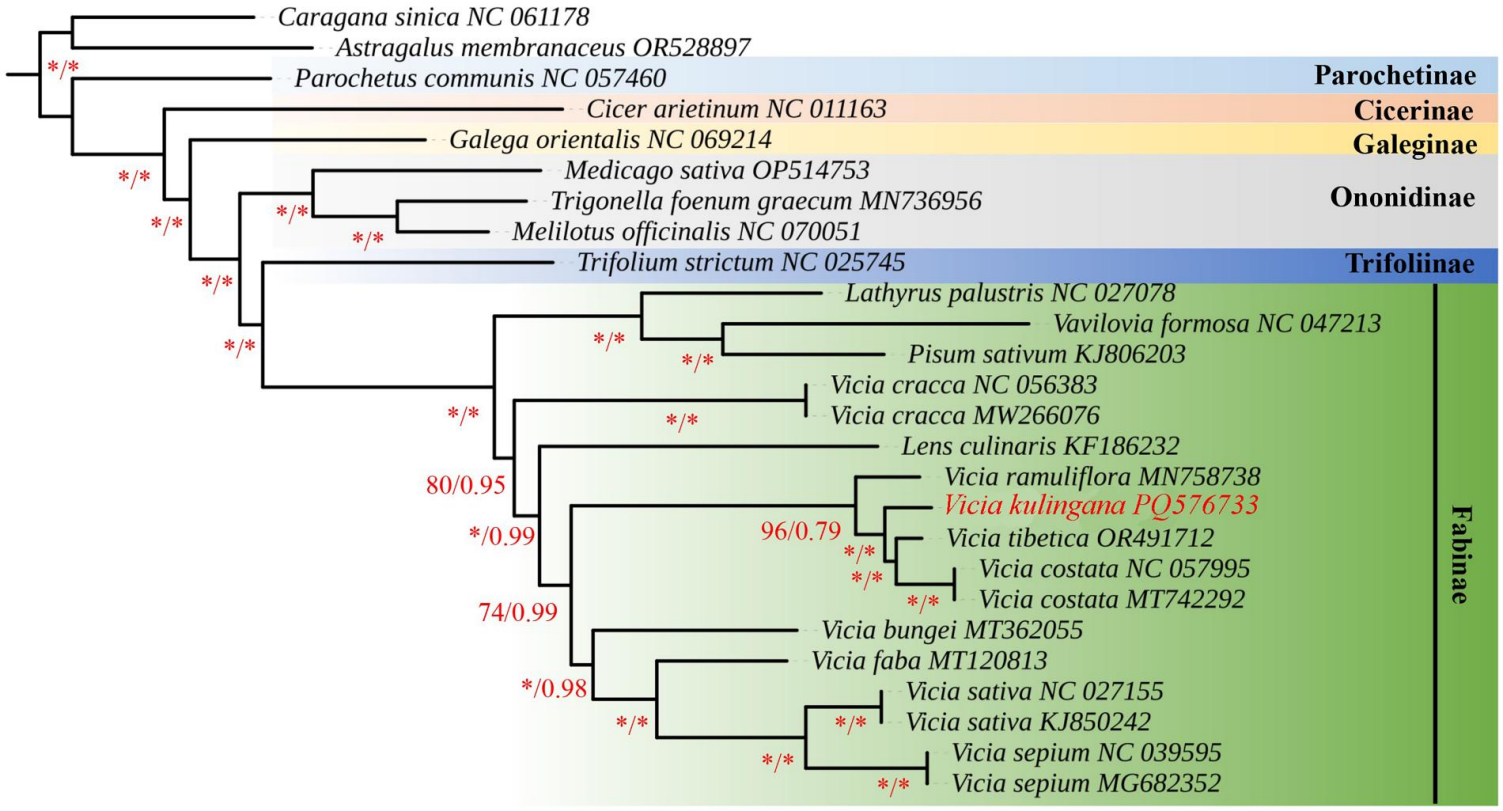
Consensus phylogenetic tree reconstructed by maximum likelihood (ML) analysis based on 56 protein-coding sequences (CDS) of 22 species, with *C. sinica* and *A. membranaceus* as outgroups. Numbers near the branches are bootstrap support (BS) percentages obtained from maximum likelihood inference. Those nodes with BS ≥ 90%, PP =1.00 were shown with asterisks. The following sequences were used: *V. ramuliflora* MN758738, *V. costata* MT742292, *V. sepium* MG682352, *V. sativa* NC027155, *V. costata* NC057995, *V. faba* MT120813, *V. sepium* NC039595, *V. sativa* KJ850242, *V. tibetica* OR491712, *V. cracca* NC056383, *V. cracca* MW266076, *V. bungei* MT362055, *cicer arietinum* NC011163, *galega orientalis* NC069214, *lathyrus palustris* NC0277078, *lens culinaris* KF186232, *medicago sativa* OP514753, *melilotus officinalis* NC070051, *parochetus cummunis* NC 057460, *Pisum sativum* KJ 806203, *astragalus membranaceus* OR528897, *caragana sinica* NC 061178, *trifolium strictum* NC025745, *trigonella foenum-graecum* MN736956, *vavilovia Formosa* NC047213. The species newly sequenced in this study is shown in red font.

Phylogenetic analysis revealed that *V. kulingana* and other three *Vicia* species (*V. ramuliflora*, *V. tibetica*, *V. costata*) are genetically closely related and are located on the same branch of the phylogenetic tree, indicating a sister group relationship among them. Interestingly, our results do not support a monophyletic group for *Vicia*, especially for the phylogenetic position of the *Lens*. More species data are needed in the future to clarify the phylogenetic relationship between the *Vicia* and the *Lens* (Figure 3).

## Discussion and conclusion

Most chloroplast genomes have a circular structure that contains a large single-copy region, an inverted repeat A region, a small single-copy region, and an inverted repeat B region. The chloroplast genomes of some legumes, including *V. sepium* and *V. bungei*, have lost one of the two inverted repeats (Li et al. 2018).

As previously reported, in this study, the chloroplast genome of *V. kulingana* lacked an inverted repeat unit. The chloroplast genome of *V. kulingana* suggests that it may belong to different evolutionary clades, such as *V. sepium* and *V. bungei*, which may share a distinct evolutionary history.

*Vicia* is mostly a climbing, trailing or creeping vine, with even pinnately compound leaves, with tendrils at the apex of the leaf axis, and most bracts are small and early falling, most without bracts. The distinction between this species and other *Vicia* species is that bracts are persistent at the base of pedicels. Although legumes’ classification depends on the morphological characters, many conflicts are encountered in this process. Many researchers have observed a morphological continuum between *Lens* and *Vicia*. Hence, *Lens* is a *Vicia* with *Lathyrus*-style characters. Moreover, *V. sativa* var. *platysperma* and *V. lunata* have an intermediate form between *Vicia* and *Lens*. Therefore, molecular tools may provide better classification and identification discrimination. Some studies have shown that *Lens* should be treated as a synonym of *Vicia* (Omar et al. 2019). Phylogenetic analysis in our study also revealed that the *Vicia* is not a monophyletic group, especially for the phylogenetic position of the *Lens*. More species data are needed in the future to clarify the phylogenetic relationship between the *Vicia* and the *Lens*.

## Supplementary Material

Figure S1.jpg

Supplement Fig2.jpg

## Data Availability

The genome sequence data that support the findings of this study are openly available in GenBank of NCBI at [https://www.ncbi.nlm.nih.gov] (https://www.ncbi.nlm.nih.gov/) under accession no. PQ576733. The associated **BioProject**, **SRA**, and **Bio-Sample** numbers are PRJNA1185893, SAMN44731224, and SRR31347921 respectively.
