# Supplementary figures and images for: Characterization and phylogenetic analysis of the chloroplast genome of Vicia kulingana (Fabaceae)
Source: Mitochondrial DNA B Resour. 2025 Mar 31;10(5):342–6. doi: 10.1080/23802359.2025.2485168 (PMC11960304; doi:10.1080/23802359.2025.2485168)

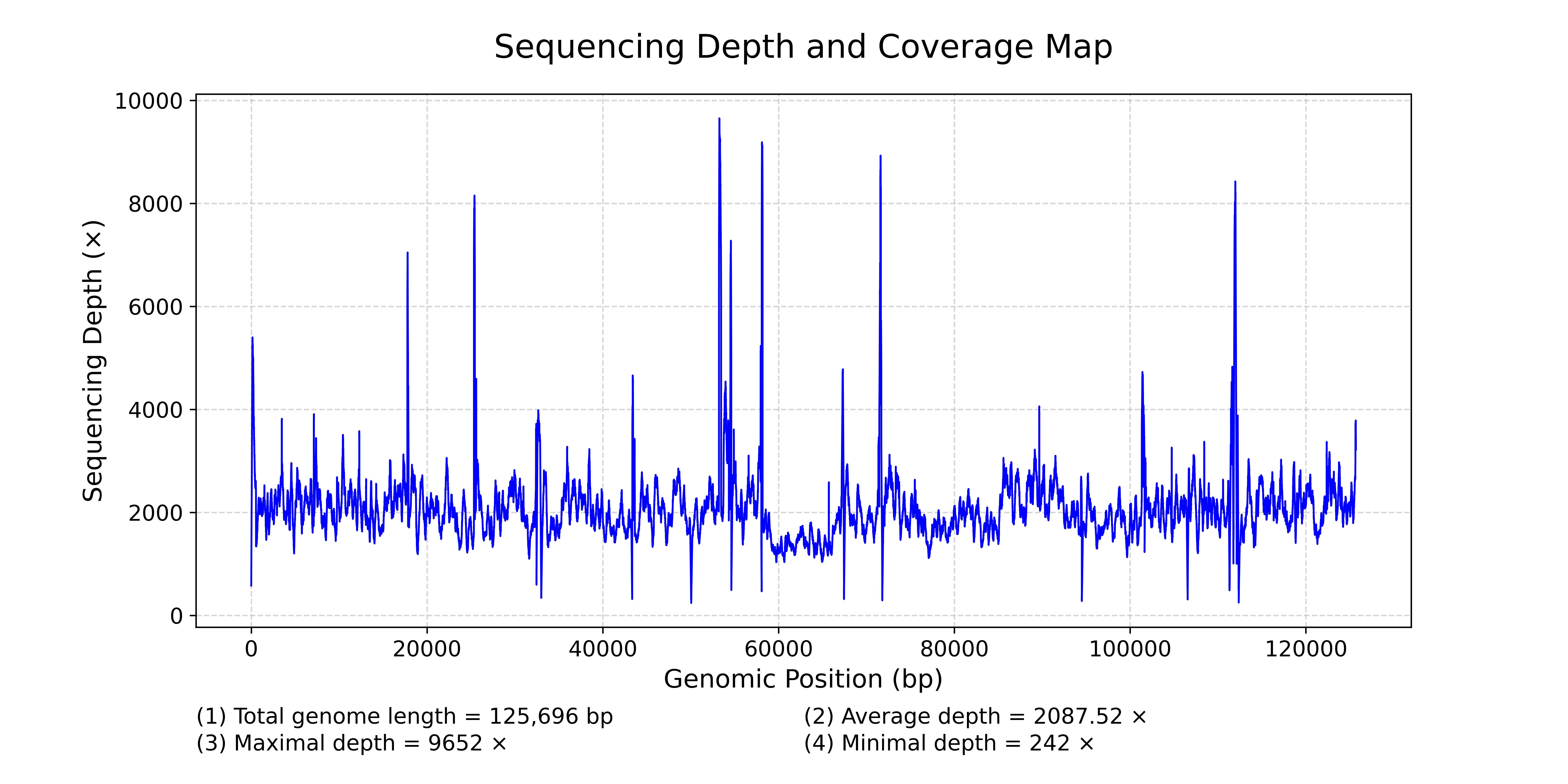

Supplement: Figure S1.jpg [file TMDN_A_2485168_SM6358.jpg]

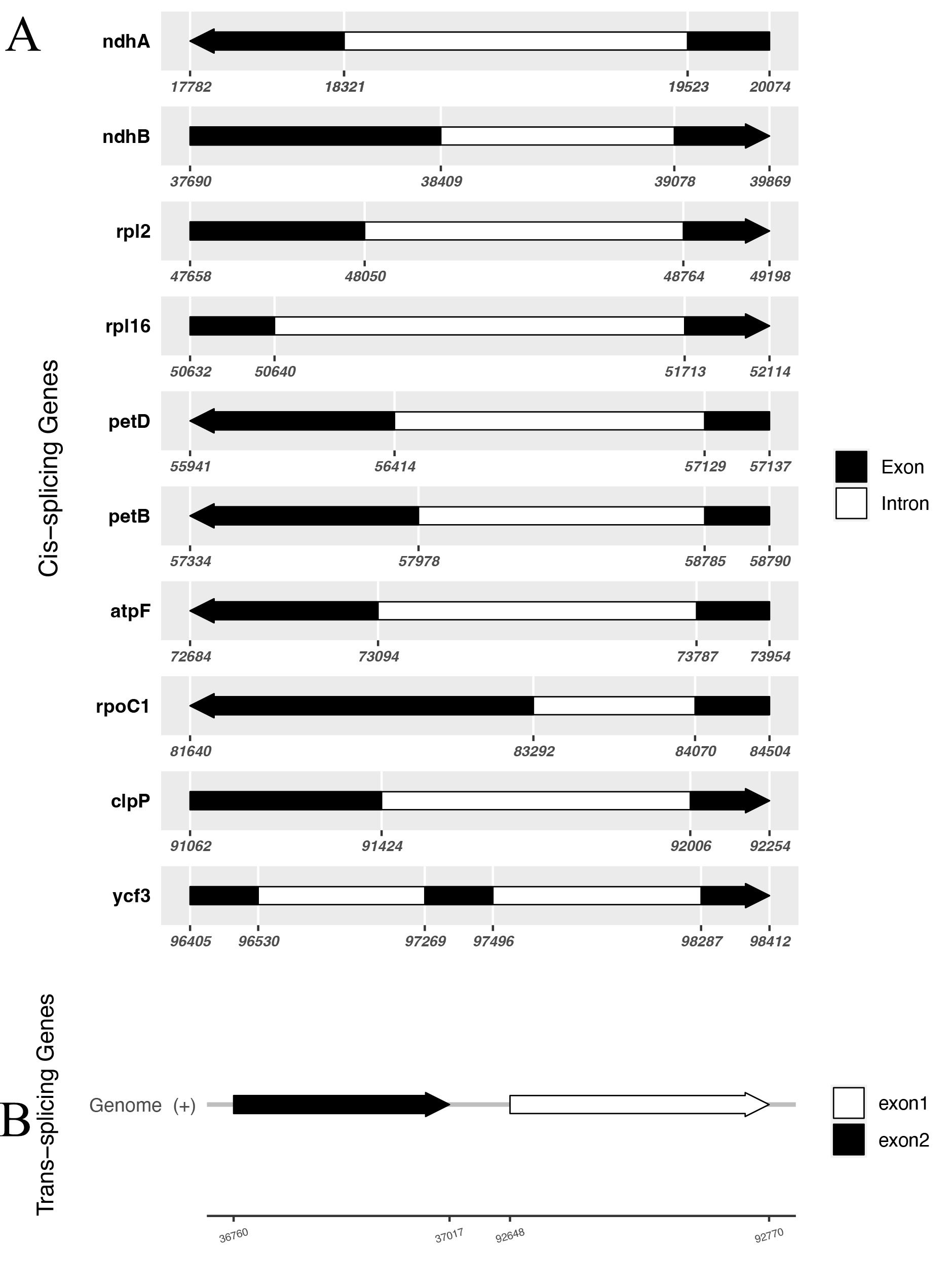

Supplement: Supplement Fig2.jpg [file TMDN_A_2485168_SM6357.jpg]
